# Treatment of Nonhealing Diabetic Lower Extremity Ulcers with Skin Graft and Autologous Platelet Gel: A Case Series

**DOI:** 10.1155/2013/837620

**Published:** 2013-03-31

**Authors:** Yuan-Sheng Tzeng, Shou-Cheng Deng, Chih-Hsing Wang, Jui-Che Tsai, Tim-Mo Chen, Thierry Burnouf

**Affiliations:** ^1^Division of Plastic Surgery, Department of Surgery, Tri-Service General Hospital, National Defense Medical Center, 11490 Taipei, Taiwan; ^2^Department of Materials Engineering, Tatung University, 10452 Taipei, Taiwan; ^3^Human Protein Process Sciences, 59000 Lille, France & Institute of Biomaterials and Tissue Engineering, Taipei Medical University, 11031 Taipei, Taiwan

## Abstract

Lower extremity ulcers in diabetic patients are difficult to treat. Recently, the use of human blood platelet-derived components in this indication has been raising interest. In this study, we have evaluated the safety and efficacy of the combination of autologous platelet gel (PG) and skin graft for treating large size recalcitrant ulcers. Eight consecutive diabetic patients aged 25 to 82 with nine nonhealing lower extremity ulcers (median size of 50 cm^2^; range 15–150 cm^2^) were treated. Skin ulcer was debrided, and the wound was sprayed after 7 to 10 days with autologous platelet-rich plasma and thrombin. Thin split-thickness skin graft with multiple slits was then applied on the wound bed and fixed with staples or cat-gut sutures. There were no adverse reactions observed during the study. Eight out of 9 skin grafts took well. The interval between skin graft and complete wound healing ranged from 2 to 3 weeks in the 8 successful cases. No ulcer recurrence was noted in those patients during the follow-up period of 2 to 19 months. In this study, the combination of autologous platelet gel and skin grafting has proven beneficial to heal large-size recalcitrant ulcers.

## 1. Introduction

About 15% of diabetic patients will develop chronic ulcer, and about 25% of those will have to undergo foot amputation [1, 2]. In the nonhealing diabetes mellitus (DM) ulcers, in addition to vascular and neurological disorders, the healing process is impaired in part due to deficiency of growth factors [3]. Becaplermin, a recombinant human platelet-derived growth factor-BB (Regranex, PDGF-BB, Systagenix Wound Management, Gargrave, UK) is the only growth factor preparation approved by the FDA for treating DM ulcers, but it requires daily applications for weeks to months [4, 5]. Live skin equivalents, known as Apligraf and Dermagraft, accelerate wound healing, but also require frequent (weekly) applications, exhibit short shelf-life, and are expensive [6]. The use of an adenovirus encoding human platelet-derived growth factor formulated in bovine collagen gel (GAM501) for treating small nonhealing diabetic foot ulcer has been reported [7, 8]. Despite these advanced researches, a more practical and effective therapy for nonhealing diabetic ulcer is clinically needed.

Platelet-rich plasma (PRP) has been proposed as an adjunct for the treatment of diabetic foot ulcers [9–11]. PRP is most often mixed with thrombin before application in order to generate a fibrin gel, often called platelet gel, and a platelet-growth-factors-rich exudate [12]. Thrombin-activated platelets release numerous growth factors from their *α*-granules [13] that can modulate cell proliferation and differentiation and accelerate soft tissue repair *in vivo* [14]. A recent systematic review and meta-analysis of the use of PRP therapy in cutaneous wounds does show that it can improve wound healing compared to control wound care in small hard-to-heal acute and chronic wounds [15, 16]. In addition, platelet materials exert antimicrobial activity against some bacteria of the skin flora [17], and clinical data show that the presence of infection is reduced in PRP-treated wounds [15]. Therefore, platelet materials exhibit a set of advantages that can provide a practical and effective treatment approach for small hard-to-heal ulcers. However, for the large unhealing diabetic ulcers, our experience is that it is imperative to use skin graft as the definite procedure for wound healing.

In a recent study with 17 ulcers of various etiology, we have shown that a skin grafting was improved by a combination of single-donor allogeneic platelet gel and fibrin glue [18]. However, as patients may express safety concerns on the use of allogeneic blood products, the current study evaluates for the first time to our knowledge the safety and efficacy of using autologous platelet gel, without fibrin glue, to enhance skin graft take for nonhealing diabetic lower extremity ulcers.

## 2. Material and Methods

### 2.1. Clinical Study Approval and Patients

This clinical study was a prospective pilot trial approved by the Institutional Review Board of Tri-Service General Hospital, National Defense Medical Center, Taipei, Taiwan (Protocol 098–05-301). Eligible patients were enrolled after informed written consent was obtained. The protocol conformed to ethical guidelines of the 1975 Declaration of Helsinki. From January 2010 to September 2012, eight consecutive diabetic patients with nine nonhealing lower extremity ulcers were treated. The ulcers were not curable for at least 3 months prior to the enrollment using conservative treatments including daily dressing change, topical application of antibiotic ointment, and synthetic dressing coverage using Aquacel and DuoDERM (ConvaTec, Garenne-Colombes, France). Pregnant women, patients with ischemic change of leg (Transcutaneous oxygen tension TcPO_2_ < 30 mmHg), severe cardiovascular disorder, and patients refusing to donate blood were excluded. No patient required revascularization surgery of the leg. TcPO_2_ was measured for vascular perfusion of the leg. All patients have value above 30 mmHg. There were two men and six women, aged 25 to 82. The ulcers had a median size of 57 cm^2^ (range 15 to 150 cm^2^). The median duration of diabetes and ulcer before study enrollment was 10.6 years (range 5 to 25 years) and 6.5 months (range 3 to 24 months). None of the patients had received conventional skin grafting in the past, and no one was tobacco user. Patient's demography is reported in Table 1.

### 2.2. Preparation of PRP

**Figure 1:**
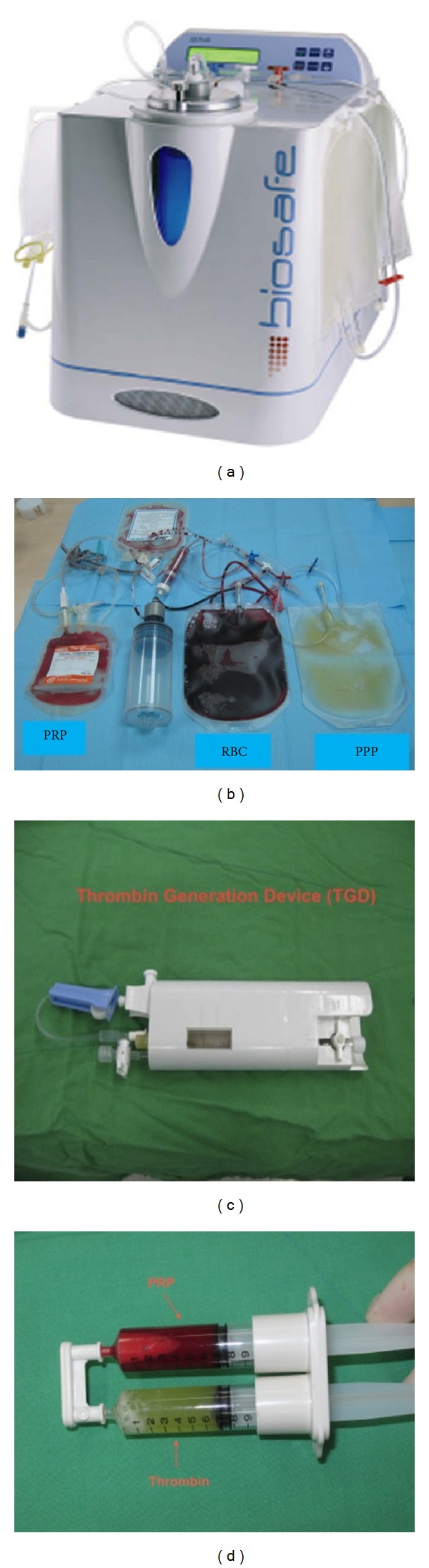
Biosafe SEPAX system (a). Autologous PRP and plasma to prepare platelet gel and thrombin (b). Thrombin generation device to activate plasma (c). Double-syringe applicator containing PRP and thrombin (d).

PRP was prepared using the SEPAX system (Biosafe SA, Eysins, Switzerland) (Figure 1(a)). An amount of 100 mL venous blood was drawn from the patient into blood bag containing 22 mL of anticoagulant (JMS Singapore Pte Ltd, Singapore). After 20 minutes of processing using the VGR protocol (SEPAX), PRP, PPP (platelet poor plasma), and RBC (red blood cell) were collected individually (Figure 1(b)). The PRP was drawn aseptically into a sterile syringe.

### 2.3. Preparation of Thrombin

Thrombin was prepared as in our previous studies [18, 19]. Then, 10 mL of PPP and 0.3 mL of a 10% calcium chloride solution were introduced into a sterile thrombin generation device (TGD-001; Merries International Inc., Shin Tien, Taiwan) (Figure 1(c)). The device was shaken gently for 30 seconds and then put aside to let the plasma activation reaction proceed at room temperature. After approximately 15 minutes, a fibrin clot was formed, and the thrombin-rich supernatant was drawn aseptically using a sterile syringe.

### 2.4. Preparation of Platelet Gel

Autologous platelet gel was obtained by spraying simultaneously equal volumes of PRP and thrombin using a spray applicator (Merries International Inc.) (Figure 1(d)). Within 5 to 10 seconds, a platelet gel was formed on the wound.

### 2.5. Surgical Procedures

The nonhealing ulcers were first debrided to remove the infected and necrotic tissues. The wounds were covered with moist saline dressing. Daily dressing change without additional treatment was performed. Repeated debridement was necessary in 6 patients because of residual necrotic tissue. The interval between the debridement and skin graft ranged from 7 to 10 days. During skin graft surgery, the wound bed was sprayed evenly with equal volumes (5 to 7 mL) of autologous PRP and autologous thrombin to form the platelet gel, and a split-thickness skin graft with multiple slits was put on the gel-covered bed, fixed with staples or cat-gut sutures, while a short leg P-P splint was used to immobilize the lower extremity. Every patient was placed on antibiotics during the course according to wound cultural results. Bolster dressing with sofa-tulle was used to avoid postgraft hematoma formation. The skin graft was checked 3 days after surgery. Negative pressure wound therapy (VAC) was not used in this study.

## 3. Results

### 3.1. Overall Clinical Data

**Figure 2:**
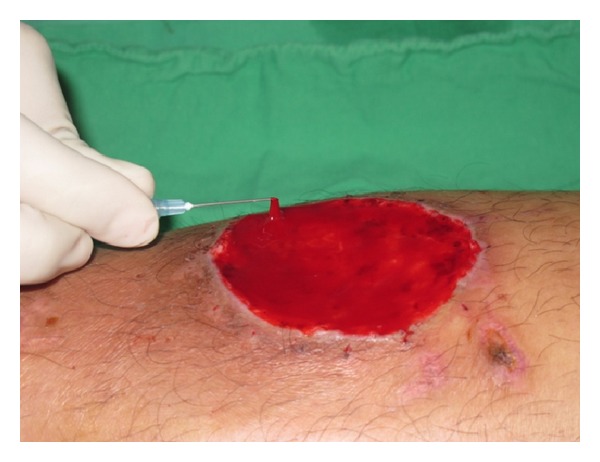
Platelet gel formed on the wound by conversion of fibrinogen into fibrin.

Fibrinogen presents in the PRP polymerized into a fibrin gel, leading to the formation of platelet gel that adhered to the wound bed (Figure 2). No treatment associated adverse reactions were observed during the study. Most (8/9) of the skin grafts took well apart from one (patient no. 7; case 3 described later). The interval between skin graft and complete wound healing in the seven successful cases ranged from 2 to 3 weeks. No recurrence of the ulcer was noted in those patients during the follow-up period, which ranged from 2 to 19 months. Review of treatments is reported in Table 1. Eight of nine ulcers had complete healing corresponding to a healing rate of 88%, the time to healing ranging from 2 to 3 weeks.

### 3.2. Case Presentations

#### 3.2.1. Case 1

**Figure 3:**
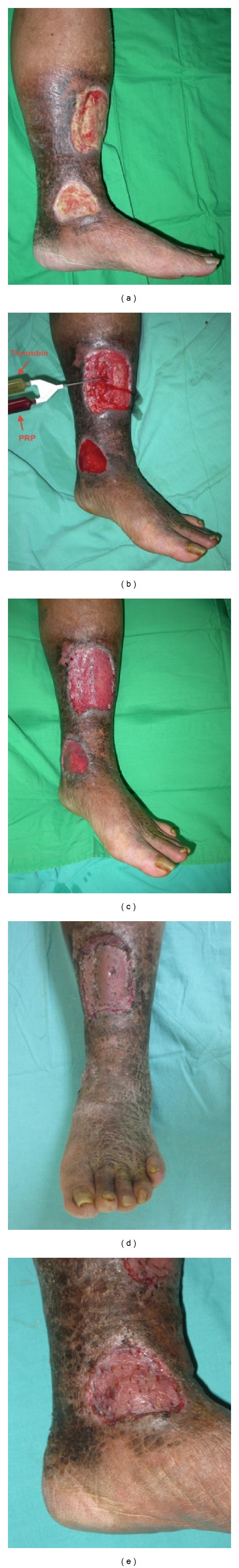
Two chronic ulcers (15 × 10 cm^2^ and 5 × 7 cm^2^) with surrounding scar tissues (a). After adequate debridement, the wound was sprayed with PRP and thrombin (b). Skin graft was applied on gel-covered wound bed (c). Durable wound coverage 10 months after skin graft (d, e).

A 65-year-old male, diabetic for 6 years, suffered from two nonhealing ulcers of left lower leg, measuring 15 × 10 cm^2^ and 5 × 7 cm^2^, respectively, due to stasis dermatitis for 6 months. The surrounding tissue was severely scared (Figure 3(a)). Debridement was performed twice to remove the necrotic tissue. One week after the second debridement, the wound bed was sprayed with autologous platelet gel (Figure 3(b)). A thin split-thickness skin graft was put on gel-covered bed (Figure 3(c)). Compression stocking (30–40 mmHg at the ankle) was used once the wound healed. The postoperative course was uneventful, and the patient has durable wound coverage 10 months after skin graft (Figures 3(d) and 3(e)).

#### 3.2.2. Case 2

**Figure 4:**
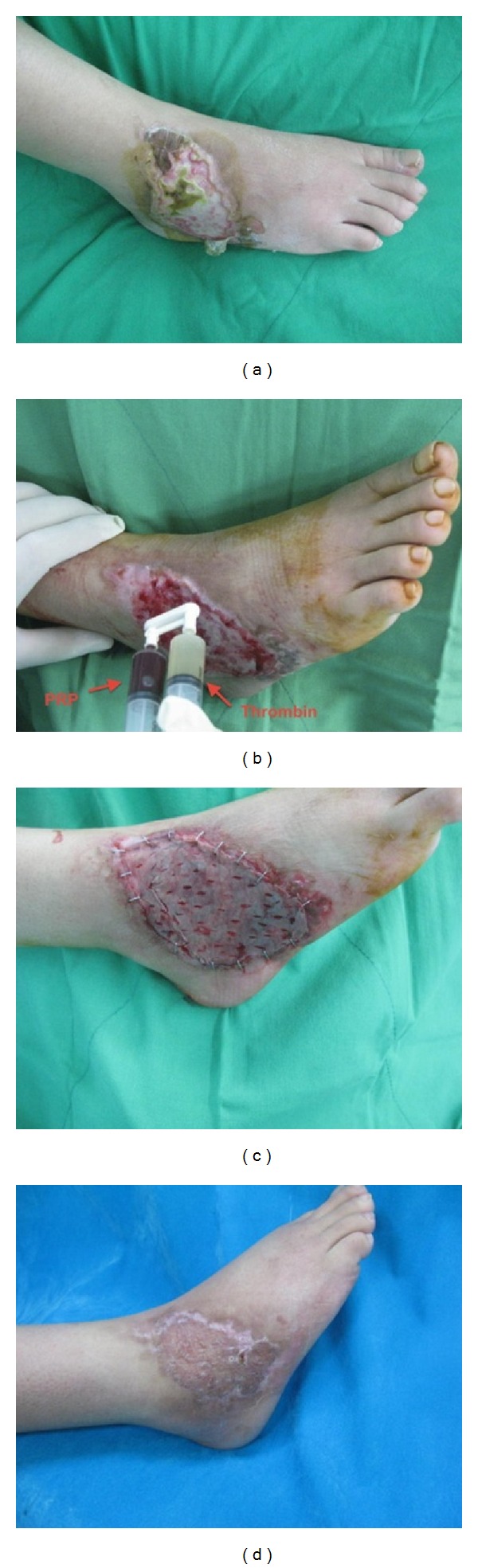
Burn injury with chronic ulcer (6 × 10 cm^2^) (a). After adequate debridement, the wound was sprayed with PRP and thrombin (b). Skin graft was applied on gel-covered wound bed (c). Durable wound coverage 12 months after skin graft (d).

A 45-year-old female, diabetic for 13 years, suffered from nonhealing ulcer over right ankle, measuring 6 × 10 cm^2^ due to infrared radiation burn for 2 months (Figure 4(a)). One week after debridement, the wound bed was sprayed with autologous platelet gel (Figure 4(b)). A split-thickness skin graft was put on gel-covered bed (Figure 3(c)). The post-operative course was uneventful, and the patient has durable wound coverage 12 months after skin graft (Figure 4(d)).

#### 3.2.3. Case 3

**Figure 5:**
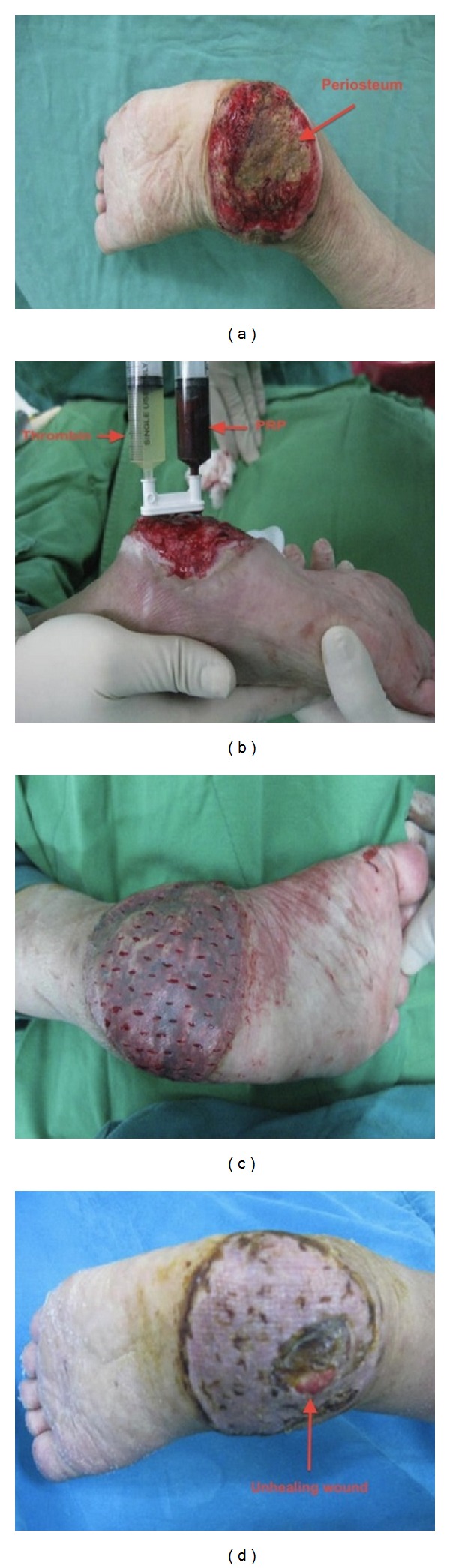
Chronic ulcer (10 × 15 cm^2^) deep to the periosteum of calcaneus bone (Arrow) (a). After adequate debridement, the wound was sprayed with PRP and thrombin (b). Skin graft was applied on gel-covered wound bed (c). Skin graft loss (3 cm^2^) over the periosteum, 2 months after skin graft (d).

A 72-year-old female, diabetic for 8 years, suffered from nonhealing ulcer over left heel, measuring 10 × 15 cm^2^, due to contusion injury for 2 months. The ulcer was deep to the periosteum of calcaneus bone (Figure 5(a)). The patient had no evidence of osteomyelitis with negative bone scan and had normal ESR (erythrocyte sedimentation rate), or CRP (C-reactive protein). Although free tissue transfer would have been required, patient refused the microsurgery, due to the age and medical condition. One week after the third debridement, the wound bed was sprayed with autologous platelet gel (Figure 5(b)). A split-thickness skin graft was put on gel-covered bed (Figure 5(c)). A skin graft loss of about 3 cm in diameter due to grafting on periosteum of calcaneus bone was noted (Figure 5(d)). The patient died 2 years after surgery because of lethal arrhythmia during dialysis. As we were told by the family, the ulcer did not heal.

## 4. Discussion

Chronic nonhealing diabetic ulcers of lower extremity develop as a result of peripheral neuropathy, ischemia, and trauma [20]. The goal of treatment is to obtain expeditious wound closure. The standard treatments include adequate debridement, control of infection, re-vascularization of ischemic tissue, and avoidance of undue pressure on the wound. Live skin equivalents show some efficacy but have short shelf-life and are expensive [6]. GAM501 was found to help the treatment of nonhealing diabetic foot ulcer in 15 patients [7]. However, the ulcer size at base-line was small (1.2 to 4,86 cm^2^), thereby questioning the clinical relevance for the cure of serious ulcer cases [7]. In a more recent study, complete closure incidence observed in GAM501 (ulcer size: 3.1 ± 1.7 cm^2^) and formulated collagen alone (ulcer size: 2.9 ± 1.1 cm^2^) was not statistically significant [8].

*In vivo* cellular-therapy-based PRP or growth factors can serve as an adjunct to those treatments. There is increasing evidence of the efficacy of PRP-based materials to enhance wound healing [15], and, in particular, the results of clinical studies using these materials to treat small-size nonhealing diabetic ulcers are definitely encouraging [18, 21, 22]. In the most common size of diabetic foot ulcers (<7.0 cm^2^ in area and <2.0 cm^3^ in volume), PRP gel-treated wounds are more likely to heal than control wounds [11].

The benefits of PRP in the treatment of severe and large ulcers have not been evaluated in randomized clinical trials [23]. From a clinical point of view, our experience is that skin grafting is commonly required as a definite surgical procedure for healing large size and deep ulcers. In a recent study, we have presented the benefits of a novel approach for leg ulcer treatment that combines three blood components (PRP, thrombin, and cryoprecipitate) from single-donor allogeneic origin with skin grafting [18]. PRP and human thrombin are first sprayed on the debrided wound to form a platelet gel, and thin spilt-thickness skin graft is then applied on top of the wound. Fibrin glue, obtained by mixing cryoprecipitate with thrombin, was sprayed on the graft to form a fibrin glue that acted as a hemostatic tissue sealant [24] that fixed the graft and avoided the use of staple or sutures [18].

In the current study, patients expressed some concerns about the use of allogeneic blood components due to perceived viral infectious risks. They were able and willing to donate about 100 mL of blood that was centrifuged in the Biosafe SEPAX system to obtain autologous PRP and PPP. PPP was activated by calcium chloride in a specifically designed medical device to generate thrombin. We could not use fibrin glue as it would have required collecting a large volume of blood (typically 450 mL) from the patients to obtain enough plasma for cryoprecipitation. In addition, preparing autologous cryoprecipitate under safe and standardized conditions is not easy within a hospital setting. After debridement of the ulcers, converting a chronic ulcer into acute wound, autologous platelet gel obtained by mixing PRP and thrombin was applied on the wound to form a platelet gel [18, 19]. A thin split-thickness skin graft was applied on top of the platelet gel and fixed with staples or cat-gut sutures. As in our previous study [18], platelet gel was found to enhance the take of the skin graft. Eight out of the 9 skin grafts took without major loss, time to healing ranged from 2 to 3 weeks, and patients achieved durable wound healing in the follow-up period, ranging from 10 to 19 months. One case presented skin graft loss due to exposure of periosteum of calcaneus bone, which would have required a more sophisticated microsurgical free tissue transfer which was denied by the patient.

PRP was prepared using a medical device that concentrates platelets 2.5-to 3.5-fold compared to baseline values in whole blood. Activation by thrombin releases multiple growth factors from the platelet alpha-granules [25]. Those include three isomers of platelet-derived growth factor (PDGF-AA, PDGF-AB, and PDGF-BB), two isomers of transforming growth factor-*β* (TGF-*β*1 and TGF-*β*2), vascular endothelial growth factor (VEGF), and epithelial growth factor (EGF). They are important for neovascularization by mesenchymal cell recruitment and extracellular matrix synthesis [25], resulting in favorable skin graft incorporation. Previous studies using different PRP production devices showed that PDGF-AB and TGF-*β*1 concentrations in platelet releasates range from 100 to 200 ng/mL [13, 26–28]. PDGF-BB is at about 10 ng/mL [26], EGF and VEGF at 1–5 ng/mL, TGF-*β*2 at about 0.5 ng/mL [26], and IGF-1 at about 100 ng/mL [29]. Such a physiological mixture of growth factors may be advantageous clinically to achieve wound healing compared to single recombinant growth factor like PDGF-BB [4, 5].

There are pros and cons in the use of autologous versus allogeneic blood materials. In the absence of pathogen inactivation treatment, a major advantage of using autologous platelet gel is avoiding the ethical and legal concerns of exposing the patient to the viral risks of allogeneic products [30], especially in countries with high infectious rates and limited donor screening and donation testing [31]. Using autologous blood leads to better acceptance of the surgical procedure by some patients. Drawbacks of autologous products include potential larger individual variability in the quality of PRP compared to allogeneic products prepared from healthy blood following standardized working procedures of blood establishments [21]. Another limitation relates to the difficulty of preparing autologous cryoprecipitate as a source for fibrin glue. Fibrin glue may be beneficial to stabilize the graft as it comes into direct contact with the wound [18] and to avoid the use of staples or sutures. Finally, preparing autologous thrombin from the patient's plasma avoids relying on bovine thrombin that may carry immunological [32, 33] and infectious risks, most particularly transmissible spongiform encephalopathy agent responsible for Creutzfeldt-Jakob disease [34]. A prospective, randomized, controlled trial of autologous platelet-rich plasma gel for the treatment of diabetic foot ulcers indicated that 13 out 19 patients (68.4%) treated with PRP gel healed in 12 weeks [11]. The effect of split-thickness skin grafts versus a conservative wound dressing on the healing times of diabetic foot ulcers has also been studied. The results showed that a 100% skin graft take was recorded in 84% of the patients on the fifth postoperative day and in 62% on weeks 3 and 8, but 8% had ulcer recurrence and 4% a superficial infection within the following year [35]. Comparing to these previous studies, our results suggest that combining PRP and skin graft enhances the efficacy of treating chronic diabetic wounds by enhancing healing rate and decreasing recurrence rate.

In conclusion, although the clinical safety and effectiveness data is derived from a pilot study rather than from a randomized controlled trial, it provides, together with our previous series [18], a confirmation of the advantages of platelet materials in skin graft procedure to treat large nonhealing diabetic ulcers of lower extremity.

## Figures and Tables

**Table 1:** Patients demography, clinical situation, ulcer location and size, and time to healing.

Patient	Age	Gender	Diabetes duration (year)	Glycated hemoglobin (%)	Cause of ulcer	Comorbidity	Ulcer location	Ulcer Size (cm)	Duration of ulcer	Take of skin graft	Time to healing	Follow-up months
1	62	F	25	10.2	Pressure sore	Renal failure; hypertension; hyperlipidemia	Right heel	4 × 7	4 months	Complete	2 weeks	12
2	25	F	5	7.1	Falling down	Rheumatoid arthritis	Right ankle	5 × 8	2 years	Complete	3 weeks	13
3	82	F	11	6.8	Cellulitis	Hypertension	Right lower leg	3 × 5	2 months	Complete	2 weeks	13
4	47	M	7	6.5	Traffic accident	Nil	Right lower leg	4 × 5	3 months	Complete	3 weeks	10
5	65	M	6	6.0	Stasis dermatitis	Varicose vein Hypertension	Left lower leg	15 × 10 5 × 7	6 months	Complete	2 weeks	10
6	80	F	10	8.2	Falling down	peripheral arterial occlusive disease Hypertension	Right ankle	3 × 5	3 months	Complete	2 weeks	19
7	72	F	8	5.5	Contusion injury	Hypertension Cervix Ca.	Left heel	8 × 10	2 months	3 × 3 cm^2^ skin graft loss	Residual ulcer	Passed away 2 years after surgery
8	45	F	13	7.7	Infrared radiation burn	Spinal cavernous angioma s/p OP with paralysis	Right ankle	6 × 10	2 months	Complete	3 weeks	18
